# Disturbed Subjective Sleep in Chinese Females with Type 2 Diabetes on Insulin Therapy

**DOI:** 10.1371/journal.pone.0054951

**Published:** 2013-01-28

**Authors:** Yingxiang Song, Xiao Ye, Leqin Ye, Bijun Li, Lijun Wang, Yanyin Hua

**Affiliations:** Department of Endocrinology, Zhejiang Provincial People’s Hospital, Hangzhou, Zhejiang Province, China; University of Porto, Portugal

## Abstract

**Objectives:**

The aim of this study is to investigate the prevalence and determine the possible risk factors of poor sleep quality in Chinese type 2 diabetes patients with insulin treatment.

**Methods:**

140 type 2 diabetes patients with insulin treatments were enrolled in our study. General characteristics and laboratory testing such glycosylated hemoglobin A1c (HbA1c), fasting plasma glucose (FPG), post-prandial plasma glucose (PPG) were measured. Every patient completed Chinese version of Pittsburgh Sleep Quality Index (PSQI) questionnaire. PSQI global score>5 was defined as poor sleep quality.

**Results:**

Global PSQI score was significantly higher in female type 2 diabetes patients with insulin treatment than male (7.52 vs 6.08, P<0.05). After adjusting for age, BMI, FPG, PPG, HbA1c and duration of diabetes, female is still an independent risk factor for poor sleep quality [OR = 2.55, 95% confidence interval (CI) = 1.24–5.27, P = 0.01].

**Conclusion:**

The results suggest that we found poor sleep quality in female Chinese type 2 diabetes patients with insulin treatment and these findings may contribute to sleep disorder control in female type 2 diabetes.

## Introduction

Diabetes is a major chronic disease with significant implications for the global society and economy. Because of rapid lifestyle changes, the prevalence of diabetes has increased dramatically in recent years [1], resulting in a serious public health issue in China. Traditional intervention strategies, including lifestyle changes such as weight loss, healthy eating, and increased physical activity, are designed to prevent and manage diabetes and its complications. However, more efforts are needed to develop new strategies. One component is to examine the relationship between diabetes and sleep quality.

Poor sleep quality and sleep disorders, particularly insomnia, are common issues in primary care [2], [3]. Early study showed that sleep debt may influence carbohydrate metabolism or endocrine [4]. In recent research, reduced insulin sensitivity and increased insulin resistance was found after short term sleep restriction [5], [6]. These results indicated short sleep duration or poor sleep quality may related to glucose metabolism. Obstructive sleep apnoea (OSA) was another sleep disorder with increasingly incidence and was considered to be a risk factor for cardiovascular diseases. In recent studies, a close connection between OSA and glucose metabolism or insulin resistance was found, and OSA was proved to be a novel risk factor for metabolic syndrome or diabetes [7]–[9]. Moreover, high prevalence of OSA was also reported in type 1 or type 2 diabetes patients compared to community group [10], [11].

Meanwhile, several recent studies have also revealed a significantly association between diabetes and sleep quality. Cross-sectional studies have shown that both IGT and diabetes are associated with short sleep [12]. Meanwhile, prospective studies have shown that short sleep duration and poor sleep quality increase the risk of diabetes in Asian people [13]. Therefore, poor sleep quality appears to be a novel risk factor for diabetes.

For patients with diabetes, sleep disturbances were correlated with glycaemic control in some observational studies. An Italian study showed that type 2 diabetes may be associated with sleep disruptions, even in the absence of complications or obesity [14]. Similarly, a study of Chinese individuals revealed that poor sleep quality were significantly correlated with inadequate glycaemic control in subjects with type 2 diabetes [15].

Insulin is a widely used antidiabetic therapy, particularly among patients with elevated HbA1c or poor glycaemic control. Exogenous insulin administration and insulin resistance was reported to be significantly correlated with sympathetic overactivity and may increase sympathetic activity [16]. Thus, insulin administration may play an important role in the prevalence of sleep abnormalities. However, few studies have examined sleep quality in patients with type 2 diabetes using insulin. Therefore, we conducted a cross-sectional study to determine the incidence of poor sleep quality in Chinese patients with type 2 diabetes using insulin therapy. Possible risk factors for poor sleep quality were evaluated using the Pittsburgh Sleep Quality Index (PSQI), a subjective sleep quality questionnaire.

## Patients and Methods

Study protocol was approved by Ethics Committee of Zhejiang Provincial People's Hospital and was conducted in accordance with the Declaration of Helsinki. Written informed consent was obtained from all participants.

Patients with type 2 diabetes using insulin therapy and who attended the diabetes clinic or outpatient centre at the Endocrinology Department, Zhejiang Provincial People’s Hospital, Hangzhou, China, between January 2012 and June 2012 were included in this study. The total study sample consisted of 140 patients.

### 1. Inclusion Criteria and Entrolment

The inclusion criteria in this study consisted of patients (1) with type 2 diabetes;(2) with insulin treatments plus or without other oral anti-diabetic agents; (3) patients with baseline clinical information and laboratory data. Exclusion criteria were as follows: (1) with type 1 diabetes or patients diagnosed with type 2 diabetes within 1 year of the study;(2) with painful diabetic sensory neuropath or other comorbidities including renal impairment, liver dysfunction, cardiovascular disease;(3) previously diagnosed sleep disorders;(4) with mental illness or use any kind of psychotropic medication;(5) working in nights shifts in the last 3 months or travelling aross time zones within 1 month;(6) with other endocrine disorders such as, thyroid disease or chronic use of glucocorticoids;(7) with alcoholism or smoking history;(8) age <18 years;(9) pregnancy or lactation.

### 2. Definitions and Study Design

Insulin treatment was defined as continuous insulin administration for the preceding 3 months, with at least one injection per day. Type 2 diabetes was diagnosed according to World Health Organization (WHO) criteria, 1999. Type 1 diabetes was defined as diabetic ketoacidosis, acute hyperglycaemic symptoms and ketonuria of ≥3, or continuous use of insulin for the first year after diagnosis. Hepatic function impairment defined as plasma aminotransferase and/or gamma-glutamyltransferase level higher than 2.5 times of upper limit. Renal dysfunction definied as serum creatinine >150 µmol/L.

Medical records were reviewed for all patients to record information on age, sex, duration of diabetes, and medication usage. Height, weight, body mass index (BMI) and laboratory tests, including glycosylated haemoglobin A1c (HbA1c), fasting plasma glucose (FPG), and post-prandial plasma glucose (PPG), were determined for all patients. Laboratory test results were collected during the study or obtained from medical records within 1 week preceding current study.

All fasting venous blood samples were drawn between 8∶00 AM to 9∶00 AM and post-prandial blood samples were drawn within 15 minutes of time points. HbA1c level was assayed using high-performance liquid chromatography (BIO-RAD Diagnositic Group, CA, USA). Plasma glucose was measured using a glucose-oxidase method (GOD/PAP, Olympus Diagnostics, Tokyo, Japan).

The PSQI is a self-completed questionnaire that assesses subjective sleep quality and disturbances over the preceding 1 month. The scale included 19 individual items, which generate seven component scores: subjective sleep quality, sleep latency, sleep duration, habitual sleep efficiency, sleep disturbances, use of sleeping medications, and daytime dysfunction. The sum of scores for these seven components yields the PSQI global score. PSQI global scores ≤5 are defined as “good sleep quality” and scores >5 are defined as “poor sleep quality” [17].

### 3. Statistical Analysis

Continuous variables were expressed as mean ± standard deviation, and categorical variables are expressed as percentages. Student’s *t*-test and the χ^2^ test were used to compare differences in the continuous and categorical variables, respectively. The nonparametric Mann–Whitney U-test were also performed as appropriate. Logistic regression models were used to analysis the relationship between PSQI global score and other variables, including age, sex, BMI, HbA1c, and duration of diabetes. Multivariate logistic regression analyses were performed to determine independent associations between these variables and good/poor sleep. The stepwise method was used, with entry and removal probabilities of 0.05 and 0.1, respectively. The odds ratios (OR) and 95% confidence intervals (CI) were determined for all variables. All analyses were performed using SPSS version 13.0 for Windows (SPSS Inc., Chicago, IL, USA). Values of *P*<0.05 were considered statistically significant.

## Results

Overall, 140 patients with type 2 diabetes on insulin therapy were included in our study. The duration of diabetes was categorized into four categories (1–3 years, 3–5 years, 5–10 years, and >10 years). The baseline characteristics of the participants are listed in Table 1. The mean ages of the total cohort, females, and males were 56.79±14.08 years, 54.79±13.69 years, and 58.17±14.27 years, respectively.

**Table 1:** Clinical characteristics of 140 patients with type 2 diabetes on insulin therapy.

Characteristics	Female	Male	Overal
	n = 57	n = 83	n = 140
Age, yr	54.79±13.69	58.17±14.27	56.79±14.08
Height, cm	158.28±4.42	167.60±8.09#	163.81±8.22
Weight, kg	56.91±9.73	69.45±11.59#	64.35±12.47
BMI, kg/m^2^	22.75±4.00	24.85±4.84#	24.20±5.46
HbA1c, %	7.79±1.65	7.90±1.62	7.85±1.63
FPG, mmol/L	8.66±3.56	8.75±3.39	8.71±3.45
PPG, mmol/L	13.27±4.93	13.02±5.12	13.14±5.03
Duration of diabetes			
1–3 years	5	12	17
3–5 years	24	30	54
5–10 years	18	23	41
>10 years	10	18	28
Global PSQI score	7.52±4.19	6.08±3.84*	6.67±4.03

Student’s t-test. female vs male *P<0.05, #P<0.01.

BMI, body mass index; HbA1c, glycosylated hemoglobin A1c; PSQI, Pitts-burgh sleep quality index; FPG, fasting plasma glucose; PPG, post-prandial plasma glucose.

The mean global PSQI score was 6.67±4.03 in the total cohort, indicating poor overall sleep quality. However, the proportion of subjects with a PSQI global score ≥5 was significantly different between males and females (39% vs 63%, *P* = 0.007) (Table 2). Univariate analysis showed that subjective sleep quality, sleep efficiency (*P*<0.05), and sleep latency (*P*<0.01) were significantly worse in females than in males (Table 3). The PSQI global score was also poorer in females (*P*<0.05).

**Table 2:** Prevalence of good/poor sleep quality in males and females.

Characteristics	Female	Male	P value
Good sleep quality	21 (37%)	50 (61%)	0.007
(PSQI score≤5)			
Poor sleep quality	36 (63%)	33(39%)	
(PSQI score >5)			

Chi-square test.

**Table 3:** Comparison of PSQI component scores between males and females.

PSQI component	Female	Male	Overall
Subjective sleep quality	1.24±0.71	0.98±0.68*	1.09±0.70
Sleep latency	1.49±0.75	1.10±0.85#	1.26±0.83
Sleep duration	1.01±0.97	0.83±0.90	0.90±0.93
Sleep efficiency	1.21±1.14	0.79±1.35*	0.96±1.28
Sleep disturbance	1.12±0.59	1.16±0.76	1.15±0.69
Hypnotic medication use	0.43±0.90	0.28±0.75	0.35±0.82
Daytime dysfunction	0.98±0.95	1.03±1.05	1.01±1.01
Global PSQI score	7.52±4.19	6.08±3.84*	6.67±4.03

Mann–Whitney U-test. *P<0.05, #P<0.01.

Table 4 shows the results of multivariate logistic regression analysis used to analyse the effects of age, sex, BMI, HbA1c, and duration of diabetes on sleep quality, as represented by the PSQI global score. Global PSQI score ≤5 was defined as good sleep quality and PSQI>5 was defined as poor sleep quality. In this analysis, only sex was an independent risk factor for poor sleep quality; the adjusted OR for females versus males was 2.55 (95% CI = 1.24–5.27, *P* = 0.01).

**Table 4:** Results of multivariate logistic regression analysis of the association between patient’s characteristics and disturbed subjective sleep.

Variable	B	SD	OR	95% CI	P value
Age	0.01	0.013	1	0.97–1.03	0.77
Sex					
Male	baseline				
Female	0.93	0.369	2.55	1.24–5.27	0.01
BMI	−0.01	0.42	0.98	0.90–1.06	0.70
HbA1c	−0.13	0.11	0.87	0.70–1.09	0.23
Duration of diabetes	0.17	0.19	1.18	0.81–1.73	0.37

Multivariate logistic regression analysis.

B, regression coefficient; SD, standard error; OR, Odds Ratio; CI, confidence interval; BMI, body mass index; HbA1c, glycosylated hemoglobin A1c.

## Discussion

Our study showed that poorer sleep quality in patients with type 2 diabetes using insulin therapy. Compared with the results of a same Chinese population-based study involving patients with type 2 diabetes, despite a lower HbA1c level (8.32% vs 7.85%), the PSQI global score was higher (6.33 vs 6.67) in patients using exogenous insulin [7]. A cross-sectional study performed in India also revealed that the PSQI global score was significantly higher in patients treated with in insulin (insulin alone or insulin plus oral antidiabetic drugs) compared with patients treated with oral antidiabetic drugs alone [18].

Obesity and exogenous insulin may contribute to these results. The mean BMI in our study was 24.20 kg/m^2^, which exceeds the threshold for overweight in China (≥24.0 kg/m^2^). A relationship between obesity and sleep quality was found in African-American individuals [19]. Similarly, other studies [20] have proved that overweight or obesity are correlated with insulin resistance in diabetes, and that insulin resistance is significantly correlated with sleep quality[21], [22]. Another possible explanation for these results is the use of exogenous insulin. As described above, exogenous insulin may play a crucial role in sleep abnormalities [16].

The PSQI global score was significantly higher in females than in males. Component scores that were significantly worse in females than in males included subjective sleep quality, sleep latency, and sleep efficiency. In further analysis, poor sleep quality was defined as a PSQI global score >5. After adjusting for age, BMI, HbA1c, and duration of diabetes, sex was an independent risk factor for poor sleep quality, with an adjusted OR of 2.55 for females compare with males. Large epidemiological studies focusing on hypertension [23] and cardiovascular disease [24] have similarly shown a sex-specific pattern in sleep abnormalities in patients with hypertension or cardiovascular disease, with worse sleep quality in women than in men. Another study of patients with diabetes has also documented worse sleep quality in females than in males [25].

The mechanisms by which sex may influence the prevalence of poor sleep quality are still unknown. The possible explanations for this phenomenon is that there are differences in peroxisome proliferator-activated receptor (PPAR)-α expression levels between males and females [26]. Because PPARα is associated with obesity, insulin resistance, and type 2 diabetes [27], this receptor may also influence sleep quality [21], [22].

This study has several limitations. First, this was a small sample cross-sectional study without long-term follow-up, we were unable to evaluation the causal relationship between gender and poor sleep quality. Second, the duration of diabetes was a risk factor for poor sleep quality in an earlier study [10] but not in our study. One explanation for this difference is that we included it as a categorical variable in our study. Third, we did not record the number of insulin doses per day, the dosage of oral anti-diabetic agents (OADs) or the prevalence of nocturnal hypoglycaemia. Therefore, it was not possible to analysis the potential associations between insulin injection frequency (particularly injections at night),usage of OADs,or nocturnal hypoglycaemia with sleep quality. We plan to conduct another study to evaluate these possible associations. Fourth, we used a questionnaire to evaluate sleep quality, rather than performing polysomnography. Therefore, the impact of other sleep disorders, such as OSA, could not be assessed.

### Conclusion

To the best of our knowledge, this was the first study to assess sleep quality in patients with type 2 diabetes on insulin therapy. Patients with type 2 diabetes and high HbA1c often require insulin therapy. We found that overall sleep quality was quite poor in this cohort of patients, and that the prevalence of sleep disorders was particularly high in women. Therefore, female patients with type 2 diabetes are more likely to develop subjective sleep disorders during insulin therapy than males. More studies are needed to examine the nature of sleep disturbances in patients with type 2 diabetes on insulin therapy, particularly among females.
